# Thickness Dependent Effective Viscosity of a Polymer Solution near an Interface Probed by a Quartz Crystal Microbalance with Dissipation Method

**DOI:** 10.1038/srep08491

**Published:** 2015-02-16

**Authors:** Jiajie Fang, Tao Zhu, Jie Sheng, Zhongying Jiang, Yuqiang Ma

**Affiliations:** 1 Collaborative Innovation Center of Advanced Microstructures and Department of Physics, Nanjing University, Nanjing 210093, China; 2 School of Electronics and Information and College of Chemistry and Biological Science, Yi Li Normal University, Yining 835000, China; 3 Laboratory of Soft Condensed Matter Physics and Interdisciplinary Research, Soochow University, Suzhou 215006, China

## Abstract

The solution viscosity near an interface, which affects the solution behavior and the molecular dynamics in the solution, differs from the bulk. This paper measured the effective viscosity of a dilute poly (ethylene glycol) (PEG) solution adjacent to a Au electrode using the quartz crystal microbalance with dissipation (QCM-D) technique. We evidenced that the effect of an adsorbed PEG layer can be ignored, and calculated the zero shear rate effective viscosity to remove attenuation of high shear frequency oscillations. By increasing the overtone *n* from 3 to 13, the thickness of the sensed polymer solution decreased from ~70 to 30 nm. The zero shear rate effective viscosity of the polymer solution and longest relaxation time of PEG chains within it decrease with increasing solution thickness. The change trends are independent of the relation between the apparent viscosity and shear frequency and the values of the involved parameter, suggesting that the polymer solution and polymer chains closer to a solid substrate have a greater effective viscosity and slower relaxation mode, respectively. This method can study the effect of an interface presence on behavior and phenomena relating to the effective viscosity of polymer solutions, including the dynamics of discrete polymer chains.

Viscosity is one of the most fundamental parameters of a material that plays an important role in industry, science, and human health^1^,^2^. Food viscosity affects food texture^1^. Fluid viscosity determines phenomena such as wetting and spreading^3^,^4^, which are ubiquitous in nature and technology. Ink viscosity affects inkjet printing^5^,^6^, as well as subsequent applications including fabricating organic transistors, light-emitting diodes, ceramics, and biopolymer arrays^5^. Solvent viscosity influences molecular behavior in the solution, e.g., the dynamics and folding of proteins^7^,^8^, processes such as metabolism, signalling, and transport of cells^9^, etc. Blood viscosity closely relates to diseases such as ischemic heart disease^10^, arterial and venous occlusions^2^, and hypertension^11^.

Because fluid viscosity significantly affects the behavior of a fluid, and molecular dynamics of molecules within it, it is of great importance to accurately measure fluid viscosity. For example, monitoring biofluid viscosity has been proposed as a diagnostic tool for detecting diseases^12^, and measuring the viscosity of dilute polymer solutions has become a simple but sensitive method to probe the conformation of discrete polymer chains^13^. Therefore, it is unsurprising that over recent decades considerable endeavor has focused on developing methods that quickly, effectively, and economically measure viscosity, explore its determinants and show how they work, and develop efficient methods for regulating fluid viscosity. This research has focused on bulk fluid, and thin films at the solid-vapour, liquid-vapour, liquid-liquid, and solid-liquid interfaces^1^,^2^,^7^,^14^,^15^,^16^,^17^,^18^,^19^,^20^. In recent years, techniques that can map local viscosity and viscosity distribution in micro- and nano-scale systems have been presented by using fluorescent dyes or gold nanoparticles as markers^9^,^21^,^22^.

However, the effective viscosity (in terms of a confined system) of a solution present on a solid substrate, as shown in Figure 1, has rarely been noted. Before contacting the solid substrate, free molecules must pass through this solution, which may undergo some property changes that can affect their behavior and properties on the substrate. This change can be probed by a viscosity measurement. The combination of the adsorbed layer viscosity, the solution adjacent to an interface (a perfect profile of the sub-solution viscosity as a function of distance from the interface), and the bulk solution will provide a clear, complete picture of how the presence of an interface changes the properties of flexible molecules.

Two aspects may be responsible for the limited interest in solutions near an interface. One is that few systems do not have adsorption at their solid-liquid interfaces, especially those with polymers and biomolecules. For these molecules, preventing this adsorption and probing the properties of the adsorbed layers are intriguing. The other possible reason is the difficulty in defining and characterizing this type of solution. In general, the properties of the solution adjacent to an interface gradually shift to the bulk solution with increasing solution thickness. Thus, a distinct interface is missing. In addition, the molecules in a solution can diffuse freely at a rate significantly greater than in a dense film. It is considerably difficult to confine the marked molecules in a solution that are adjacent to an interface, or to effectively eliminate the signal attenuation of the covering bulk solution. Moreover, the limited number of techniques that can probe thin film viscosities^14^,^16^,^21^, and the local viscosity of polymer melts and cells^9^,^21^,^22^ may not be valid. As a result, it remains unclear how the viscosity of a solution adjacent to an interface and the dynamics of molecules in such a solution are affected by the presence of the interface.

The quartz crystal microbalance with dissipation (QCM-D) technique is a universal analytical tool that has been widely employed to probe the mass, viscoelastic properties, and conformation of adsorbed molecules at solid-liquid interfaces^15^,^17^,^19^,^20^,^23^,^24^,^25^,^26^,^27^,^28^,^29^, and the viscosity of simple fluids adjacent to an interface^30^. Recently, based on an in-depth understanding of this technique^31^,^32^, we successfully utilized it to measure the effective viscosity and shear modulus of a polymer solution in the presence of an interface^33^,^34^,^35^. The results showed that in the semi-dilute regime, the specific value of *u* in the relation *η* ~ *c^u^* is equal to, or significantly smaller than, those of a bulk solution with a low (e.g., 1 k) or high (e.g., 5 k) molecular weight, respectively, where *η* and *c* are the viscosity and concentration, respectively. The difference seen in the high molecular weight solution was attributed to the presence of physical adsorption^35^.

In this paper, using a similar method, we measured the effective viscosity of a dilute 20 k molecular weight (*M*_w_) poly(ethylene glycol) (PEG) solution adjacent to an interface. There are several differences between this paper and previous studies. First, this paper focuses on the effective viscosity of a PEG solution in a dilute range. In this case, the effective viscosity is dominated by the discrete polymer chain dynamics. Previous studies focused on the effective viscosity of a semi-dilute solution. Second, herein we tackle the dependence of solution viscosity on solution thickness, which ranges from 30 to 70 nm. Previous studies dealt with the dependence of effective viscosity on concentration. Third, this paper uses a zero shear rate effective viscosity. Finally and most important, we demonstrated that the adsorption of PEG chains onto a gold surface is considerably weak at the dilute limit. Therefore, the high shear frequency oscillation^15^,^19^,^20^ and adsorbed PEG layer^35^ effects on the calculated effective viscosity of a dilute PEG solution adjacent to an interface are removed. In this instance, it is possible to study the effect of the interface presence on the viscosity of the polymer solution and the dynamics of its polymer chains, resulting from the dependence of solution viscosity on solution thickness.

## Results and Discussion

### Solution thickness in the presence of an interface measured by QCM-D

**Figure 1:**
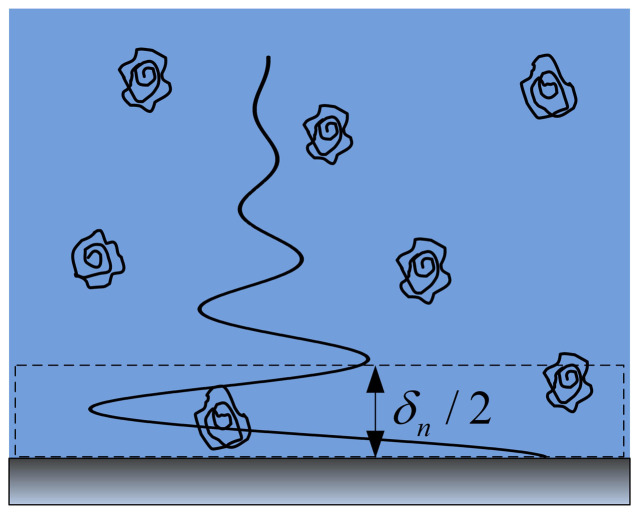
Sketch of a polymer solution adjacent to an interface (system framed by the dashed lines). Its concentration is independent of distance from the solid substrate. In this paper, the equivalent thickness of this solution, sensed by the QCM-D technique, is represented by *δ_n_*/2, which is tens of nanometers.

First, we provide an illustration of the solution adjacent to an interface. When no adsorption and depletion occurs at a solid-liquid interface, the energy of a shear acoustic wave fully dissipates in a semi-infinite solution (Figure 1). The part of the solution that is sensed by the wave is the subject of examination in this paper.

Occasionally this solution is confused with the bulk solution, as it is assumed semi-infinite. Nonetheless, please note that the actual solution thickness sensed by the QCM-D technique is roughly equivalent to *δ_n_*/2 (see Supplementary section S4), i.e., half the penetration depth of the shear acoustic wave in the solution^36^,^37^. Table 1 shows the value of *δ_n_* for water at 25 °C and a resonant frequency (*f*_0_) of 5 MHz. As the overtone of the frequency (*n,* the ratio of the crystal thickness to half the wavelength of the shear acoustic wave, which can be 3, 5, 7, 9, 11, or 13) increases from 3 to 13, the *δ_n_* decreases from 137.6 to 66.1 nm.

It is known that the properties of a solution adjacent to an interface gradually approach that of the bulk solution with increasing solution thickness. An accurate determination of the critical thickness value is difficult. However, the value of other similar cases can be used as a reference. Viscosity and other parameters, e.g., the glass transition temperature, differ significantly from the bulk for thin polymer films at solid-air or liquid-air interfaces, between a solid substrate and a thick overlay, and in a polymer matrix, as shown in Supplementary Figure S1, for films thinner than 100 nm or several to ten times the radius of gyration (*R*_g_) of the polymer (Supplementary Table S1).

As *δ_n_*/2 is significantly smaller than 100 nm, the effective viscosity of a polymer solution next to an interface sensed by the QCM-D may deviate from the bulk viscosity. Only two research groups have ever noted this deviation (Supplementary section S3). However, the solution is assumed to be viscous, not viscoelastic. In addition, the viscosity is proposed to be shear frequency and solution thickness independent (Supplementary section S3). In this paper, we attempted to remove the shear frequency oscillation effect and reveal the dependence of the solution effective viscosity (as well as the polymer chain dynamics in this solution) in the presence of an interface on solution thickness.

### Adsorption and depletion

The prerequisites of this work are no slip, no adsorption, and no depletion. Slipping leads to the failure of Equations 5 and 6, which are the basis of this paper. A detailed description of the no slip condition can be found in Supplementary section S4. In this section, we only use the “no adsorption and depletion” condition.

**Figure 2:**
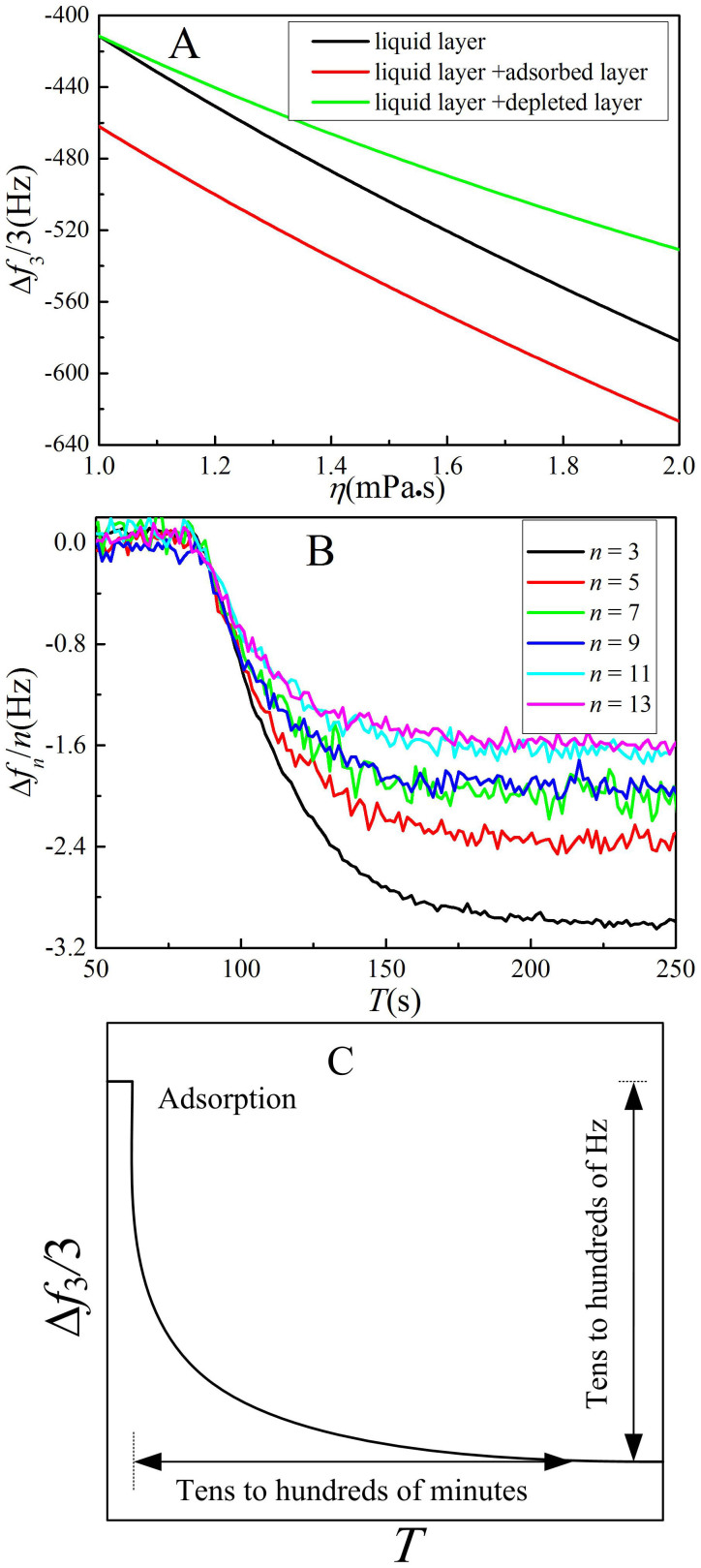
Adsorption and depletion. (A) The effects of adsorption and depletion layers on Δ*f*_3_/3 as a function of the viscosity of the solution near an interface, estimated using the Voight model^36^,^37^. The viscosities of the adsorbed and depleted layers were fixed at 10 and 1 mPa·s, respectively, and the layer thickness was 10 nm. (B) Variation of Δ*f_n_*/*n* with time with injecting a 1.3 mg/mL PEG solution. (C) Sketch of the Δ*f*_3_/3 response to the adsorbed polymer film at the solid-liquid interface. Detailed experimental examples can be found elsewhere^19^,^25^.

An adsorption and depletion, which would lead to a step-like density profile and have significant effects on the QCM-D signals (Figure 2A), should be avoided if possible. In this section, we mainly discuss adsorption, since it occurs more frequently.

The most direct and convincing route for checking adsorption and depletion is to take a density profile. These can be obtained by techniques such as neutron scattering^38^. However, such techniques require expensive equipment, which are not practical for many researchers. There are other indirect but facile methods for determining whether the effect of an adsorbed layer can be neglected.

First, there should be no substantial adsorption or depletion above the coil layer, since the interactions between the polymers and solid substrate rapidly decrease with increasing distance from the substrate. A large difference between the QCM signal of a polymer solution adjacent to an uncoated surface and a surface coated with a coil polymer brush corresponds to a strong interaction between the polymer and substrate, while a minimal difference corresponds to weak interactions. In a previous article, we modified a gold surface with 5 k thiol-PEG. In the semi-dilute regime, the values of *v* in the relation *η* ~ *c^v^* were 1.44 and 1.49 for a 20 k PEG solution adjacent to unmodified and modified gold surfaces, respectively^29^, indicating a negligible adsorption of the PEG chains on a gold surface.

Second, the QCM-D signal, Δ*f_n_*/*n*, of a layer adsorbed at a very dilute concentration generally ranges from tens to hundreds of Hz (Figure 2C). For example, the Δ*f*_3_/3 of a 10 k *M_w_* polyacrylamide layer adsorbed from an extra dilute (c*/300) solution onto a silver surface was about −30 Hz (where c* is the overlap concentration)^19^, and that of a 20 k *M_w_* poly(*N*-isopropylacrylamide) adsorbed from a 2.5 part per million (ppm) aqueous solution onto a gold surface was nearly −20 Hz^25^,^26^. In this work, we measured the Δ*f_n_*/*n* from water to a PEG solution at 1.3 mg/mL (of ~c*/20, where c* ≈ 27 mg/mL^39^); the Δ*f*_3_/3 was −3 Hz, as shown in Figure 2B. This suggests that in the dilute region, the adsorption of PEG chains on a gold surface is weak.

Third, the dilute limit requires tens to even hundreds of minutes for adsorption to reach equilibrium (Figure 2C)^19^,^25^. For a PEG solution, this time is less than 3 min (Figure 2B and Supplementary Figure S3). As the chamber volume and flow rate are 40 μL and 100 μL/min, respectively, 3 min is the expected time for new fluid to completely replace the original fluid.

The common characteristic of the above methods is that Δ*f* can be replaced with the signal of a technique sensitive to the mass at solid-liquid interfaces. Besides these, four other methods also prove a condition of no or negligible adsorption (Supplementary section S6); they are not shown because they are based upon the specific characteristics of QCM signals.

### Zero shear rate viscosity: high frequency shear effect

Before discussing the effective viscosity of a polymer solution adjacent to an interface and its dependence on solution thickness, please note that this viscosity should be a zero shear rate effective viscosity to avoid the attenuation of high frequency shear oscillations. Then, a relation between the apparent viscosity and shear frequency must be assumed, as the QCM measures at tens of millions of Hertz.

A general relation is still lacking, due to the relations in terms of the microstructure, type of applied stimuli, solvent quality, concentration, etc^13^,^40^,^41^. However, in this paper, since we deal with a specific case, we can choose a specific relation. Additionally, it is permissible to employ different relations to investigate their effects on the zero shear rate effective viscosity.

In the case of a high shear frequency oscillation, the relations between the apparent viscosity and shear frequency, according to the Zimm (Equation 1) and Rouse models (Equation 2)^13^ and Carreau equation (Equation 3)^19^,^42^, are (Supplementary section S8): 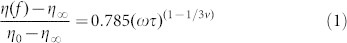

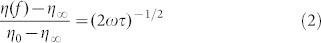

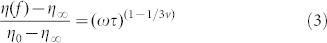
Where *η*_∞_ is the viscosity at an infinite shear rate (estimated as the solvent viscosity, *η*_b_), *η*_0_ is the zero-shear rate viscosity, and *v* is the coefficient in *R*_g_ = *aN^v^*, where *R*_g_ and *N* are the radius of gyration and degree of polymerization, respectively, and a is the size of repeated unit, *ω* = 2π*f* is the angular frequency. Using a static light scattering technique, the experimental results revealed that PEG in the molecular weight range of 6 k to 10 M, and in water at 298 K, *v* = 0.58^39^. The longest relaxation time *τ* is defined by Equation 4: 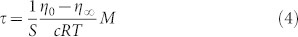
Where *S* is the coefficient relating to the distribution of relaxation times, *R* and *T* are the gas constant and temperature, respectively. The value of *S* varies from approximately 2.369–2.387 for polymers in theta solvent to values close to the Rouse value (1.645) in good solvent^41^.

In previous studies, fitting viscosity data to the Carreau model produced *τ* values three times greater than the theoretical values^42^. This is consistent with the prediction that the dynamics of a polymer chain close to a surface are slower than the dynamics in the bulk^43^. Therefore, in this paper, values of 3*τ*, *τ* and *τ*/3 are used to reveal the effect of time on the calculated zero shear rate viscosity.

### Zero shear rate effective viscosity, longest relaxation times, and their thickness dependences

**Figure 3:**
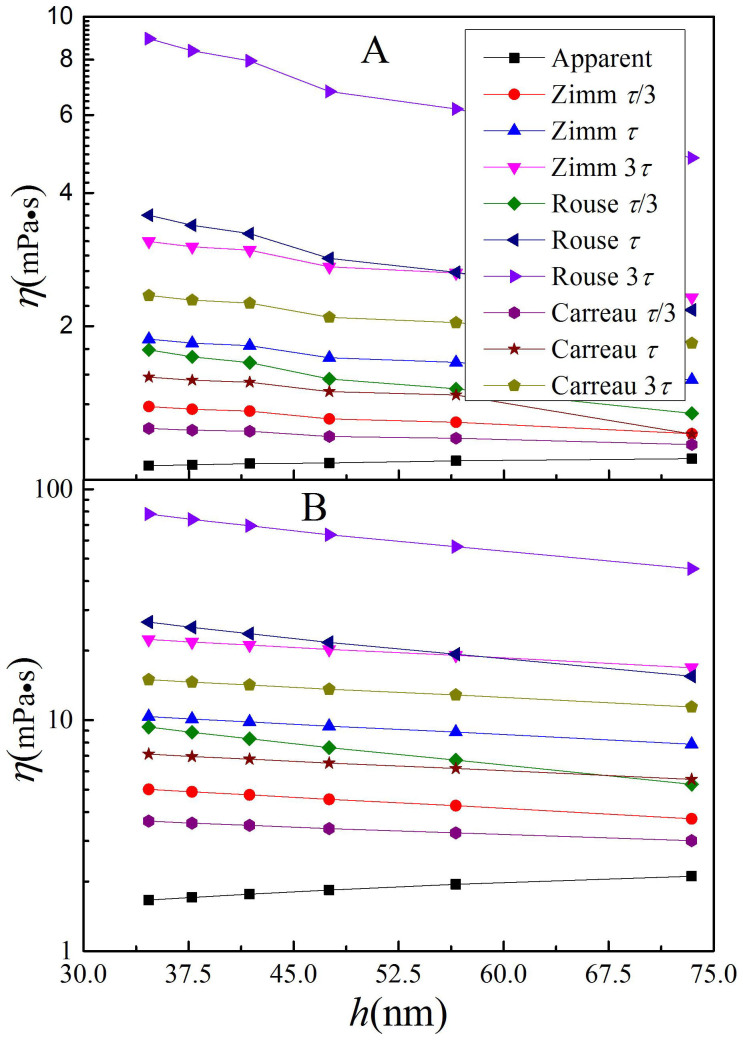
Measured apparent and calculated zero shear rate effective viscosities. The concentration is (A) 9.2 and (B) 92 mg/mL.

Figure 3 shows the apparent and zero shear rate effective viscosities calculated using Equations 1–3, at concentrations of 9.2 and 92 mg/mL, respectively. The values at other concentrations have similar dependences on the solution thickness and the employed models, and thus are not shown.

The value of *η*_0_ calculated from the Rouse mode was the largest, and that from the Carreau equation is the smallest. It increases with increasing longest relaxation time. These all agree with what was shown in Supplementary Figure S5.

The values of *η*_0_ from different equations and different relaxation times are all significantly greater than the apparent viscosity value. This can be easily understood from the view of *τ* = (20000 × 0.036 × 0.89)/(1.645 × 298.15 × 8.314) μs = 0.157 μs, where [*η*] = 4.33 × *M*^0.679^ × 10^−5^ mL/mg = 0.036 mL/mg^39^. This value closely matches experimental results measured by other techniques^44^.

**Figure 4:**
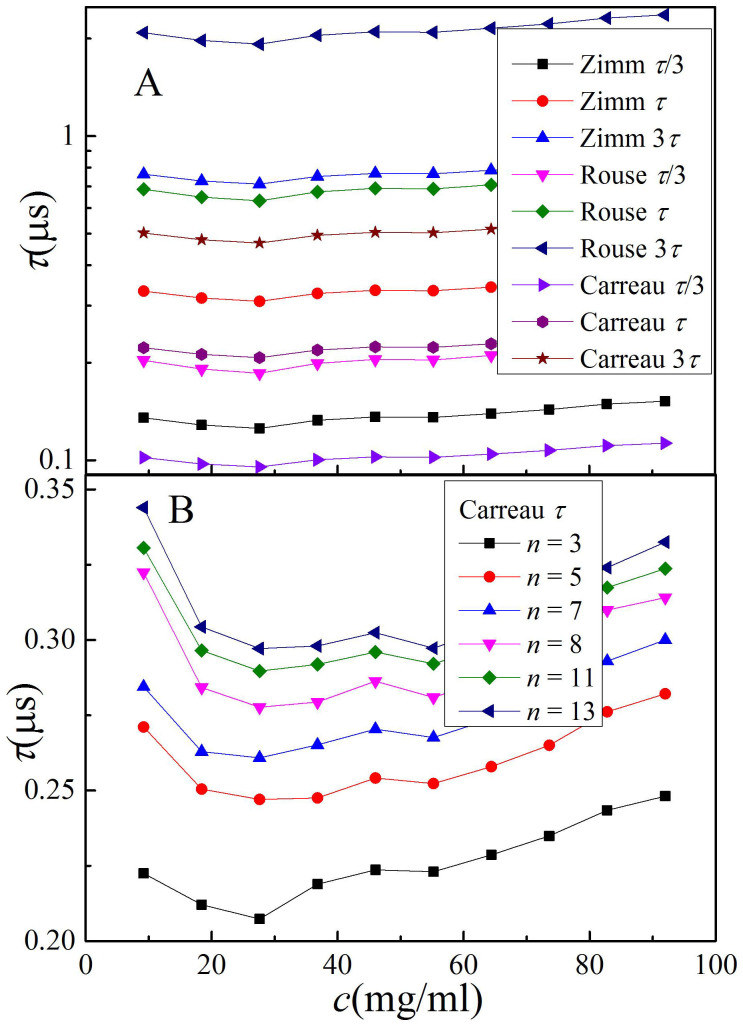
The values of the longest relaxation time at different situations. The overtone in Figure 4 (A) is third, and the equation for Figure 4 (B) is Carreau equation. All data is calculated using Equation 4.

The fitted *τ* at the third overtone is shown in Figure 4A. In all conditions, except the 3*τ* of the Rouse model, *τ* lies on the scale of 0.157 μs, the theoretical value. In the range of the measured concentration, the *τ* shift is generally less than 10%, suggesting that the polymer chains in a dilute polymer solution near an interface remain in the discrete state, although the calculated zero shear rate effective viscosity is considerably larger than 2*η*_b_.

Figure 4B shows the dependence of the *τ* value on overtone at 9.2 mg/mL. The *τ* increases with increasing overtone, i.e., decreasing solution thickness, implying that a polymer chain closer to an interface has a slower relaxation process. This is consistent with reports on the mobility of polymers near a solid substrate^43^,^45^,^46^,^47^,^48^,^49^.

**Figure 5:**
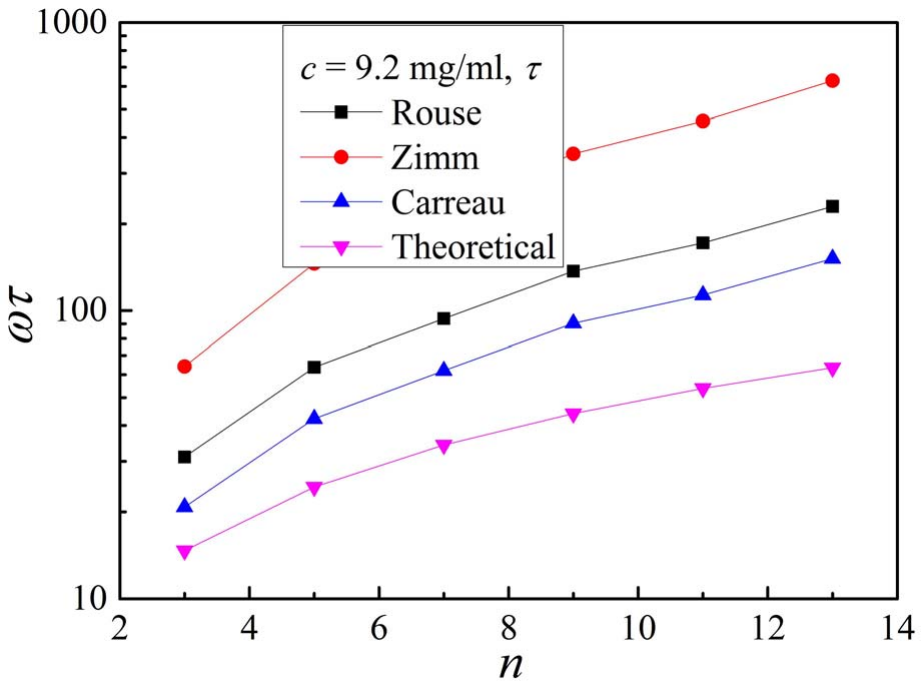
Dependence of *ωτ* calculated at 9.2 mg/mL on overtones and models. The longest relaxation time is estimated using Equation 4.

Figure 5 shows the dependence of *ωτ* calculated at 9.2 mg/mL on overtone. With the increase of the overtone from 3 to 13, the theoretical value of *ωτ* increases from about 15 to 60, and the calculated value can be as high as 630. A larger value of *ωτ* indicates a greater effect of shear thinning, implying a larger value of zero shear rate effective viscosity. This is the reason why the apparent viscosities are significantly smaller than the calculated zero shear rate effective viscosities.

The agreement between the *τ* experimental values with the theoretical value, and the small dependence of *τ* on concentration, suggest that although the specific values of *η*_0_ and *τ* depend on the models employed, the common change trends are above these models and the value of *τ*. One of these trends is that the zero shear rate effective viscosity of the solution near an interface decreases with increasing solution thickness, indicating that the sub-solution closer to the interface has a larger viscosity. However, the conclusion given by the apparent viscosity is opposite from this.

The trend in the change of the zero shear rate effective viscosity is more reasonable. From previous results, the effective viscosity of a polymer solution adjacent to an interface is expected to decrease with increasing solution thickness, because of the weakening of the effect induced by the interface presence.

These types of effects for polymer films at solid-vapour and liquid-vapour surfaces, and within a polymer matrix, have been extensively studied both theoretically and experimentally (Supplementary section S3). The results indicated that they strongly depend upon the conditions studied and the parameters measured (Supplementary section S3). In addition, different material behaviors were observed with different techniques. For example, the magnitude of *T*_g_ depression of free standing polymer films in Brillouin light scattering and ellipsometry experiments differs greatly in hole growth and lateral force microscopy experiments^50^.

The origins of these effects on different conditions are also different. The case of a solution in the presence of an interface (Case F in Supplementary Figure S1) is similar to that of a polymer film located between a solid substrate and thick overlay (Case C in Supplementary Figure S1), or a polymer film placed in a polymer matrix (Case E in Supplementary Figure S1). Among the measurements of different parameters in these two cases, the measurement of the diffusion rate intrigued us. The *R*_g_ value of PEG used in this paper is 6.715 (0.0215*M*^0.58^) or 6.654 (0.181*N*^0.583^) nm^39^, the solution thickness measured by the QCM-D ranges from about 5 to 10 *R*_g_. A neutron scattering technique revealed that the diffusion rate of polymer chains at the marked and unmarked polymer film interface (Case C in Supplementary Figure S1) increases with increasing distance of the interface from the solid substrate, approaching the bulk value when the distance is greater than 5 *R*_g_^47^. The dynamic second ion mass spectroscopy (DSIMS) results showed that the diffusion rate of polymer chains in a polymer matrix (Case E in Supplementary Figure S1) is over one order of magnitude slower than the bulk value when its distance from the solid substrate was less than 10 *R*_g_^48^.

The nature of different zero shear rate effective viscosities of a dilute polymer solution near an interface may not be identical to the nature of different diffusion rates of polymer chains in a thin polymer film, because of the different dynamics between the discrete polymer in a dilute solution and one entangled in a dense film. However, considering similar experimental situations, the *R*_g_ dependent deviation (e.g., the viscosity of the oligomer solution sensed by the QCM-D technique is the same as the bulk^35^), and several *R*_g_ or longer range effects, a discussion of the latter can be used as an excellent reference for exploring the nature of the former.

In previous discussions, long-range forces between diffusing polymer chains and the substrate, such as van der Walls forces, were not expected to be significant due to the large distances from the interface. They were then interpreted from the view of polymer-surface interaction mediated by matrix polymers^48^, solid-like or glass-like dynamics due to the effect of contact with a solid substrate^48^, effect of the techniques used^47^, etc.

The polymer-surface interaction significantly alters the dynamics of polymer chains that directly contact the solid substrate. In a polymer film, the polymer chains entangle with one another; the dynamics of one polymer chain depends strongly on the dynamics of the surrounding polymer chains. This is the reason why the polymer-surface interaction effect can persist up to several times the radius of gyration in a polymer film. However, considering the special case in this paper, the effect of this interaction can be excluded. This is because: (1) the solution in this paper is dilute, thus the dynamics of one polymer chain does not affect surrounding polymer chains, and (2) the solution concentration has a longitudinal homogeneity, thus the number of polymer chains that directly contact the solid substrate and also have different dynamics to those far away from the substrate is negligible compared with the total number of polymer chains in the solution sensed by the QCM-D technique. As the QCM-D technique measures the average dynamics of polymer chains in the solution near an interface, probing the specific dynamics of polymer chains that directly contact the solid substrate is beyond the reach of the QCM-D.

Further measurements are required to determine whether it is solid-like dynamics or conformation change or some other facts that result in a decreased effective viscosity of the polymer solution adjacent to an interface with increasing solution thickness. One candidate is to probe the relation between the effective viscosity and solution concentration and molecular weight. For dilute polymer bulk solution, solution viscosity increases linearly with increasing concentration, and the linear coefficient (intrinsic viscosity, [*η*]) increases exponentially with increasing molecular weight (*M*), [*η*] = *KM^a^*. It is the Mark-Houwink-Sakurada equation. The values of *a* and *K* depend on the particular polymer-solvent system, and the relation between the value of *a* and the conformation of the discrete polymer chain has been established very well^51^,^52^.

**Figure 6:**
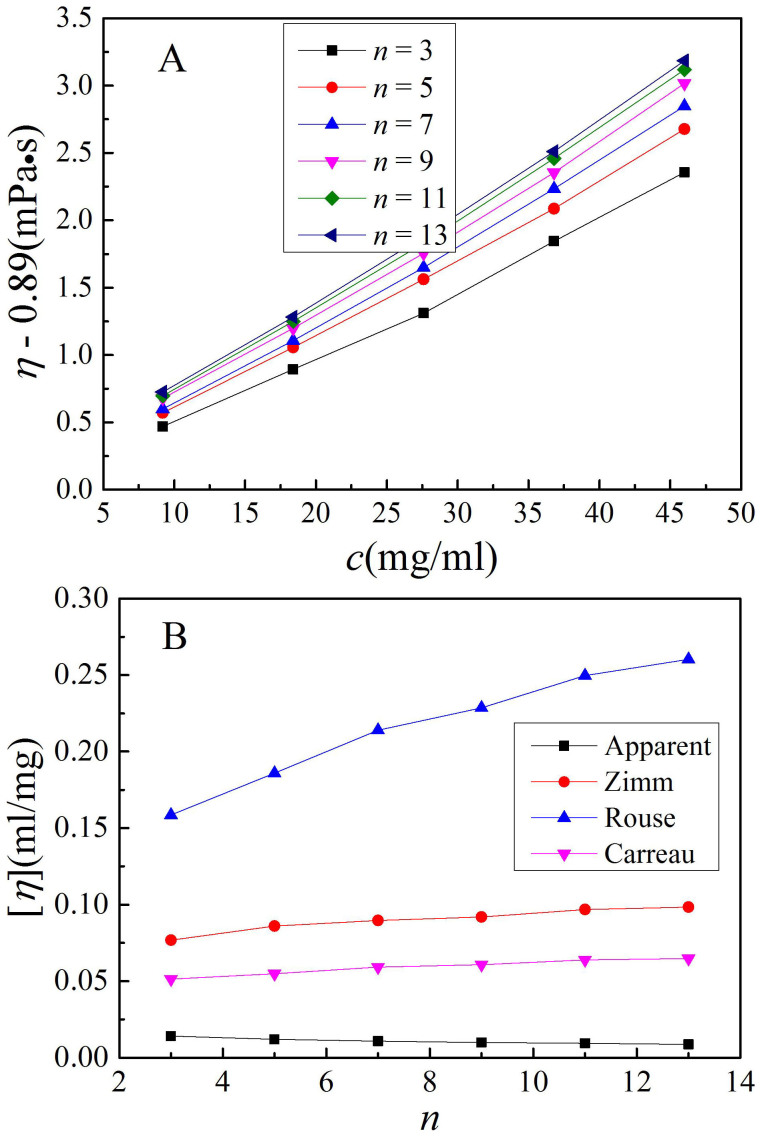
Intrinsic viscosity of a polymer solution in the presence of an interface. (A) Dependence of the dilute solution viscosity, calculated from the Carreau equation of *τ* under different overtones, on concentration. (B) Dependence of intrinsic viscosity, calculated from a linear fit of a viscosity-concentration relation, on overtone.

For a dilute polymer solution adjacent to an interface, the effective viscosity also increases linearly with the concentration, as shown in Figure 6A. Figure 6B shows the zero shear rate effective intrinsic viscosity from the Zimm and Rouse models and the Carrear equation, all of *τ*. In this instance, *τ* = [*η*]*η*_∞_*M*/(*RTS*). If the difference between the viscosity of the solution next to an interface and that of the bulk solution comes from the change in chain conformation, it is expected that [*η*] = *KM^a^* and *τ* = *KM*^1+*a*^*η*_∞_/(*RTS*). The values of *K* and *S* in two solutions remain constant, since they depend on the polymer chain dynamics. The dependence of the *a* value on solution thickness reveals the effect of an interface presence on the conformation of a discrete polymer chain. In contrast, the viscosity difference in two situations is resulted from the solid-like dynamics effect. It is unsurprising that this effect increases with increasing molecular weight. In this instance, the Mark-Houwink-Sakurada equation is no longer valid. From Equation 4, we can directly obtain the intrinsic viscosity of a dilute polymer solution near an interface, and the ratio of this intrinsic viscosity to that of the dilute bulk solution. The dependence of this ratio on the molecular weight and solution thickness can be used to comprehensively describe the solid-like dynamics effect.

## Conclusion

In summary, by using a quartz crystal microbalance with dissipation technology, we measured the effective viscosity of a polymer solution in the presence of an interface. We proved that the contribution from an adsorbed layer can be ignored, and calculated the zero shear rate effective viscosity to eliminate the effect of high shear frequency oscillations.

For a dilute PEG solution with a 20 k molecular weight, as the thickness of the solution decreases, the zero shear rate effective viscosity, and the longest relaxation time of the polymer chains increase, indicating that the sub-solution and polymer chains closer to the interface have a greater viscosity and a longer relaxation time, respectively. These change trends are above the employed relations between the apparent viscosity and shear frequency, and the chosen specific values of the involved parameters. They are consistent with the diffusion rate of polymer chains placed in a thick polymer matrix, or in a film with a thick covering layer, which increases with increasing distance to the solid substrate.

Further work will establish the relationship between effective viscosity, effective intrinsic viscosity and other parameters, such as molecular weight, to comprehensively extract the effect of an interface presence on the viscosity of a polymer solution and on the dynamics of polymer chains within the solution, finally revealing the nature of this effect.

## Methods and Experimental Sections

### Theoretical models and equations

Quartz crystal microbalance relies on the converse piezoelectric effect of quartz crystal. An alternating voltage is applied to the surfaces of an AT-cut quartz crystal via thin gold film electrodes to drive crystal resonance. In a liquid phase, the QCM equipment provides the frequency at which the resonance occurs (*f*) and the dissipation factor (*D*), a parameter characterizing the decay speed of the quartz crystal oscillation amplitude when the driving voltage is switched off (or other parameters of an identical physical meaning)^36^,^37^. The shifts of *f* and *D* are dominated by the properties (e.g., the density, thickness, viscosity, and shear modulus) of the medium above the sensor surface. In the case of a longitudinal homogeneous solution (Figure 1), the shifts are described by Equations 5 and 6,^36^,^37^: 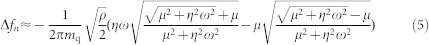

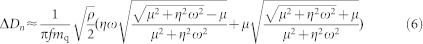


Where *m*, *ρ*, and *μ* are the mass, density, and shear modulus, respectively, the subscript q refers to the quartz crystal.

A detailed description of the QCM-D signal expressions can be found elsewhere^33^,^34^,^35^,^36^,^37^. If the medium is a Newtonian liquid, in which *μ* ≪ *ηω*, Equation 5 reduces to the well-known Kanazawa-Gorden equation (Supplementary section S4)^30^.

Equations 5 and 6 are the equations used in this paper. Although there are three unknown parameters, the solution density is nearly equal to that of the solvent when the solution near an interface is dilute.

### Materials and solution preparation

A stock solution was prepared using purified water (minimum resistivity 18.2 MΩ) from a Milli-Q Water System. Typical experimental procedures include the following steps. First, 4 g of PEG (molecular weight 20 k) was added to 40 mL of water, producing a solution concentration of ~92 mg/mL. The solution was gently stirred overnight with a magnetic stirrer at room temperature to ensure a complete dissolution. It was then mixed with purified water in volume ratios of *x*:(10 *- x*), using whole number *x* values from 1 to 10. Thus, the concentrations of the final solution were varied in regular intervals from 9.2 to 92 mg/mL. The volume of the mixed solutions was 4 mL. The solutions were gently stirred for 2 h at room temperature. To produce the dilute PEG solution, 0.5 mL of a 9.2 mg/mL solution was further diluted with 3 mL water and stirred.

### QCM measurement

An AT-cut quartz crystal sensor with a fundamental resonant frequency of 5 MHz and 14 mm diameter was purchased from Q-sense AB. The two otherwise clean sensor surfaces were coated with Au thin films to act as electrodes. The root-mean-square roughness of the resultant surfaces was less than 3 nm. Prior to use, the sensors were immersed in a 50 °C piranha solution (three parts 98% H_2_SO_4_ with one part 30% H_2_O_2_) for 5 min, then thoroughly rinsed with Milli-Q water several times.

Measurements were conducted on a Q-Sense E1. First, a consistent QCM-D baseline was obtained in water. A 1.3 mg/mL solution was then pumped into the QCM-D chamber. After confirming that the Δ*f_n_*/*n* and Δ*D_n_* approached equilibrium values quickly and with small values, a higher concentration solution was pumped into the QCM-D chamber. The measurement period for each concentration was 8–10 min. All experiments were performed at 25 ± 0.01 °C. The flow rate was 0.1 mL/min. Frequency shifts were measured with ±1 Hz precision.

## Author Contributions

F.J., Z.T., S.J., J.Z. and M.Y. prepared and performed the experiment, F.J. and J.Z. wrote the manuscript, F.J., Z.T., S.J., J.Z. and M.Y. reviewed the manuscript.

## Supplementary Material

Supplementary Information Thickness Dependent Effective Viscosity of a Polymer Solution near an Interface Probed by a Quartz Crystal Microbalance with Dissipation Method

## Figures and Tables

**Table 1:** Dependences of the *δ_n_* of water at 25 °C (*ρ* ≈ 0.997 × 10^3^ kg/m^3^, *η* ≈ 0.89 mPa·s, *f*_0 _ = 5 MHz) on overtone (*n*)

*n*	3	5	7	9	11	13
*δ_n_* (nm)	137.6	106.6	90.1	79.5	71.9	66.1
