# Supplementary material for: Thickness Dependent Effective Viscosity of a Polymer Solution near an Interface Probed by a Quartz Crystal Microbalance with Dissipation Method
Source: Sci Rep. 2015 Feb 16;5:8491. doi: 10.1038/srep08491 (PMC4329548; doi:10.1038/srep08491)
Supplement: Supplementary Information — Thickness Dependent Effective Viscosity of a Polymer Solution near an Interface Probed by a Quartz Crystal Microbalance with Dissipation Method [file srep08491-s1.doc]

Supporting Information for

Thickness Dependent Effective Viscosity of a Polymer Solution near an Interface Probed by a Quartz Crystal Microbalance with Dissipation Method

Jiajie Fang^1^, Tao Zhu^1^, Jie Sheng^2,1^, Zhongying Jiang^2,1^ & Yuqiang Ma^1,3^

^1^Collaborative Innovation Center of Advanced Microstructures and Department of Physics, Nanjing University, Nanjing 210093, China, ^2^School of Electronics and Information and College of Chemistry and Biological Science, Yi Li Normal University, Yining 83500, China, ^3^Laboratory of Soft Condensed Matter Physics and Interdisciplinary Research, Soochow University, Suzhou 215006, China

Correspondence and requests for materials should be addressed to J. Z. Y. ([jiangzhying@163.com](mailto:jiangzhying@163.com)) or Y. Q. M. ([myqiang@nju.edu.cn](mailto:myqiang@nju.edu.cn))

**S1. Figure S1**. A simple summary of the situations made for studying the properties of nanoscale film. A: supported or free standing film, B: labeled film with a film underlayer, C: labeled film sandwiched between a solid substrate and an overlayer, D: film with a liquid underlayer, E: labeled film placed in a polymer matrix, or with two layer on each side, F: boundary solution sandwiched between a solid substrate and a bulk liquid, the case investigated in this paper.


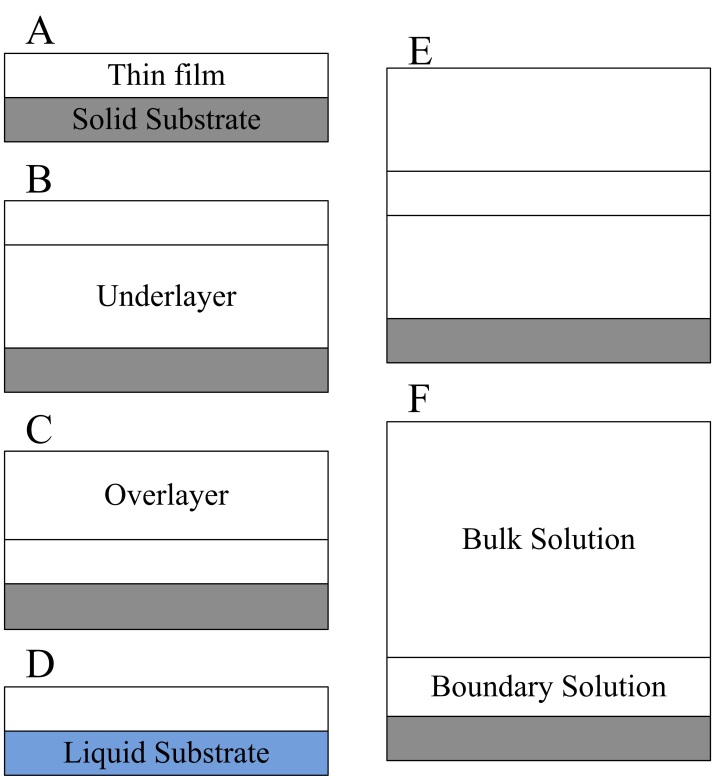


**S2. Table S1**. The major conclusion of the studies on the properties of thin films under the situations listed in Figure S1

|  | Properties of thin films and macromolecules in these films |
| --- | --- |
| A | With increasing film thickness, in generally *T*_g_ of supported (non-interaction) or free-standing polymer film decreases, and of film on an attractive substrate increases^3^  the magnitude of the *T*_g_ depression depends on the techniques used^3^  No direct link to molecular weight was found, and generally no effect is reported for films of thickness greater than100 nm^3^ |
|  | FRAPP results showed that *D* of PS films on patterned quartz disks decreases by more than a factor of 2 when the film thickness is less than ~ 150 nm, 50 times of *R*_g_^4^ |
|  | Monomer - monomer exclude volume interactions of thin layer of entangled polymers predicted an exponential increase of the viscosity as layer thickness decreases^5^ |
|  | XPCS results showed that the surface viscosity of PS film of about 130 nm thick is about 30 % lower than the rest of the film^6^ |
| B | FI results showed that the shift magnitude of *T*_g_ of polymer films depends on species and thickness of investigated polymer film and underlayer^7,8^ |
| C | For *T*_g_ measurement, the same as Case B^7,8^ |
|  | DSIMS results showed that *D* of PS chains near melt-solid interface for PVP and SiO covered surfaces were smaller by respectively ~ 3 and ~ 100 than that near the vacuum interface^9^ |
|  | NR results showed that *D* of PMMA chains at the interface increases to the bulk value for its distance to silicon substrate greater than about 4 or 5 *R*_g_^10^ |
| D | ST results showed that viscosity increases with thickness and approach the bulk value for film greater than about 10 *R*_g_^11^ |
| E | For *T*_g_ measurement, the same as Case B^7,8^ |
|  | DSIMS results showed that *D* of PS chains in PS matrix increases with increasing distance from the solid substrate, and is one order magnitude slower than bulk at the distance 10 *R*_g_^12^ |
|  | FI results showed that the structural relaxation of PMMA is reduced by a factor of 2 and by a factor of 15 for enough thin overlay and underlay, respectively^13^ |

Note: *T_g_*: glass transition temperature, *D*: diffusion coefficients, FRAPP: fluorescence recovery after patterned photobleaching, XPCS: x-ray photon correlation spectroscopy, FI: fluorescence intensity, DSIMS: dynamic second ion mass spectroscopy, NR: neutron reflectrometry, ST: surface tension

**S3. Two Past Studies of the Effect of Solution on QCM Signals**

Munro et al. fitted the experimental data of the adsorption of Polyacrylamide (PA, molecular weight 10 k and 1 M) from aqueous solutions onto gold and silver surfaces with six different models, Newtonian liquid, Non-Newtonian liquid, elastic film, viscous film, viscoelastic film, and viscoelastic film + Non-Newtonian liquid^1^. For controlling the number of fitting parameters less than five, shear modulus of PA solution was fixed at zero, and viscosities of adsorbed film and boundary solution were assumed to be frequency independent. For 1 M PA, the experimental Δ*f_n_* and Δ*D_n_* values match quite well with values from viscoelastic film and viscoelastic film + Non-Newtonian liquid models. For 10 k PA, the experimental Δ*f_n_* and Δ*D_n_* cannot match with values of all six models. The authors also pointed out that, in the range of shear frequency from 5 to 35 MHz, viscosity of 10 k PA solution decreases dramatically, while viscosity of 1 M PA solution approaches the solvent. The assumption of frequency independent viscosity of boundary solution may be one of the reasons leading to the discrepancy for 10 k PA.

Bordes et al. provided three methods to separate the bulk effects and bound mass: measuring the density and the viscosity of the solution to achieve the expected values of Δ*f* and Δ*D* (equation S1 and S2, as shown below); generating a calibration curve on a nonadsorbing surface; estimating Δ*f* of bulk solution from the formula Δ*f* = -*f*Δ*D*/2 (equation S3)^2^.

The first and third methods are based on the assumptions that the bulk solution is a Newtonian liquid, and Δ*D* of an adsorbed film is very low. As stated in that paper, in the case of polymer solution, one may question whether these preconditions remain effective. In such cases, the first method fails because viscosity and shear modulus of such solution sensed by QCM technique may differ from those obtained with other rheometers, since QCM measures at frequencies that exceed those employed in most conventional bulk rheometers by several orders of magnitude. The third method loses effectiveness additionally because Δ*D* of a viscoelastic film is not very low.

The second way, which is very straightforward, remains valid. The difficulty is to find a surface on which there is no or negligible adsorption.

**S4. Accuracy Estimation, No-Slip Examination, and Calibration.** We discuss no-slip condition because slipping leads equations 5 and 6, which are the basis of our method, to fail.

The most direct and persuasive method to prove the no slip condition is to investigate the oscillation velocity of the sensor surface and contacting layer. However, this method was negated by its complexity. While a simple and feasible method is to compare experimental QCM-D signals of a system of standard viscosity, with theoretical values^14,15^. Indeed slip is not the only factor contributing to the deviation of calculated viscosity from the theoretical, and it is hard to discriminate the contribution of slip from others, but an excellent agreement between experimental viscosity data and the standard is taken to confirm a no slip condition and as an implication of highly reliable data.

The simplest samples having the definite viscosity are common solvent, such as water, methanol, ethanol, or their mixtures. For these samples *μ* << *ηω*, equations 5 and 6 reduce to

$\Delta f_{n}\approx-\frac{1}{2\text{π}m_{\text{q}}}\sqrt{\frac{\rho\eta\omega}{2}}$ (S1)

$\Delta D_{n}\approx\frac{1}{\text{π}nf_{0}m_{\text{q}}}\sqrt{\frac{\rho\eta\omega}{2}}$ (S2)

where *m*, *ρ*, *η* and *μ* are the mass, density and viscosity, and shear modulus, respectively, *f*_0_ is the fundamental resonant frequency of quartz crystal, *ω* = 2π*nf*_0_ is angular frequency, *n* is overtone. Equation S1 is the Kanazawa-Gordon equation, which describes how common solvent and small rigid molecule solution change the resonant frequency of quartz crystal^14^.

From equations S1 and S2, we have

$\Delta f_{n}=-\frac{nf_{0}}{2}\Delta D_{n}$ (S3)

Equation S3 clearly shows that, there is a simple relation between Δ*f_n_* and Δ*D_n_* of a Newtonian liquid. It is also the criterion to adjust whether the fluid is Newtonian^1^.

From equations S1 and S2, we know that Δ*f_n_*/*f* = (*ρη*/2*ω*)^1/2^/*m*_q_ = *ρδ_n_*/2*m*_q_. This shows that the traditional Sauerbrey equation still remain valid, with the mass within the regime *δ_n_*/2. Then, *δ_n_*/2 can be regarded as the thickness of the solution film sensed by QCM-D technique.

Figure S2 shows Δ*f_n_*/*n* and Δ*D_n_* from vapor to milli-Q water (25 °C), the calculated viscosities, and the deviations from the reported value from literature (0.89 mPa⋅s). Small deviations (less than 2 %) imply a lack of slipping.

Furthermore, we calculated the viscosity of solution film of other simple fluids such as ethanol-water mixtures with different ethanol: water volume ratios. These fluids led to the same conclusions.


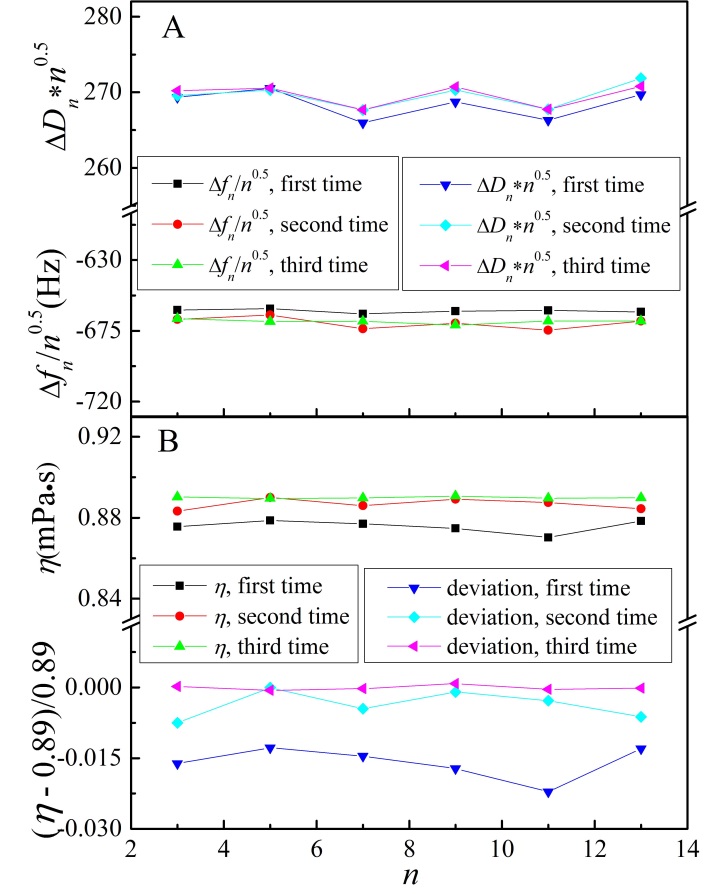


**Figure S2**. (A) Dependence of Δ*f_n_*/*n*^0.5^ and *n*^0.5^Δ*D_n_* on overtone *n*. (B) Calculated viscosity and the deviation from the accepted literature value (0.89 mPa**⋅**s), for water at 25 °C. For boundary solutions of simple fluids, Δ*f_n_*/*n*^0.5^ and *n*^0.5^Δ*D_n_* should be independent of *n*.

To reduce the effect of the small deviation (2% or less) on the following calculation, we replace the experimental QCM signals from gas phase to water with the theoretical signals. In this case, Δ*f_n_* and Δ*D_n_* used to calculate viscosity and shear modulus of boundary solution can be described as^16-18^

$\Delta f_{n}\approx-63.75\sqrt{\rho\eta nf_{0}^{3}}+\Delta f_{\text{water}\to\text{solution}}$ (S4) $\Delta D_{n}\approx31.88\sqrt{\rho\eta f_{0}/n}\times10^{-6}+\Delta D_{\text{water}\to\text{solution}}$ (S5)

Such replacement can be regarded as a calibration which is experimentally very simple. The closer the viscosity and density of the fluid being examined is to the referenced fluid, the more precise this method is.

**S5. Figure S3.** Time dependences of (A) Δ*f_n_*/*n* and (B) Δ*D_n_* of PEG solution film with different concentrations. The legend in Figure S4A also applies to 4B. As a new solution of higher concentration was injected, Δ*f_n_*/*n* and Δ*D_n_* approached new equilibrium values quickly.


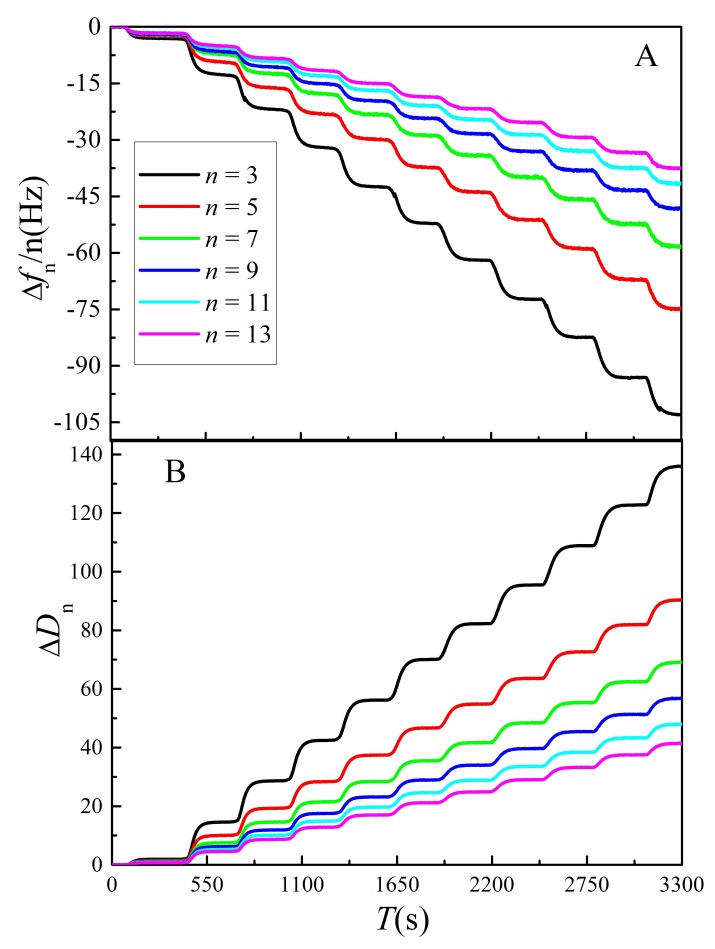


**S6. Four other aspects proving the condition of no or negligible adsorption.**

First, for quartz crystal with a fundamental frequency of 5 MHz, the value of Δ*D_n_*/(-Δ*f_n_*/*n*) of adsorbed layers is generally smaller than 0.2 × 10^-6^,^1,19-26^ and of a Newtonian liquid is 0.4 × 10^-6^.^16,27,28^ For adsorbed layer and solution film, this value decreases and increases with increasing shear modulus, respectively^16^. It is 0.63 × 10^-6^ for a 1.3 mg/mL PEG solution film, meaning that the dilute polymer solution film is non-Newtonian. This is consistent with previous observations that dilute bulk polymer solution is non-Newtonian^29^.

Second, it is known that Δ*f* ~ *n* for a solid film, and Δ*f* ~ *n*^0.5^ for a Newtonian liquid^1,27,28^. For 10 k *M*_w_ polyacrylamide adsorbed from dilute (*c*^*^/30) solution onto gold and silver surfaces, the coefficients are 0.83 and 0.87 (prerinse), and 0.97 and 1.07 (postrinse), respectively^1^. From values shown in Figure 2B, this coefficient is 0.565 for a 1.3 mg/mL PEG solution film. A large deviation from 1 implies that the adsorption is very weak.

Third, the specific relation between Δ*f_n_*/*n*, Δ*D_n_* and concentration can be used to adjust the contribution magnitude of adsorbed layer. In general, the equilibrium QCM signals of adsorbed layer approaches the plateau at a concentration much smaller than *c*^*1,22,23^. However, in this paper the QCM signals are proportional to the concentration, as shown in Figure S4A and S4B, and Table S2 (The time dependence of original experimental data can be found in Figure S3). This suggests that the signals are dominated by the solution film.

Fourth, the dependence of Δ*D_n_*/(-Δ*f_n_*/*n*) on concentration also can be used to adjust whether the adsorption can be ignored. If both solution film and adsorbed layer contribute to Δ*D_n_* and Δ*f_n_*/*n*, the slope of Δ*D_n_* - (-Δ*f_n_*/*n*) curve would vary with concentration because Δ*f_n_*/*n* and Δ*D_n_* of solution film and adsorbed layer have different dependences on the concentration**.** However, Δ*D*_3_ increases proportionally with -Δ*f*_3_/3 (Figure 2C). Similarly, this also indicates a neglected depletion.


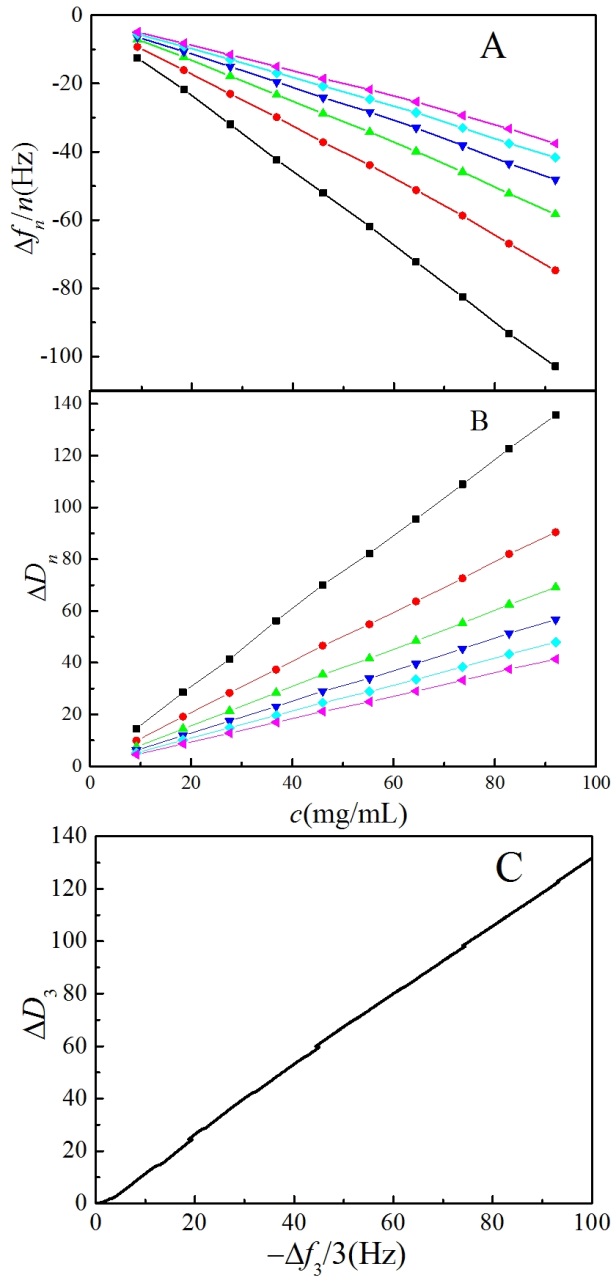


**Figure S4**. (A) and (B) Dependence of Δ*f_n_*/*n* and Δ*D_n_* from PEG solution film on solution concentration. From black, red, green, blue, cyan, to magenta, the overtone is 3, 5, 7, 9, 11, and 13, respectively. (C) A plot of Δ*D*_3_ versus -Δ*f*_3_/3 of PEG solution of different concentrations.

**S7. Table S2**. Results of the linear fitting of the relation between Δ*f_n_*, Δ*D_n_* and concentrations (Figure S4A and S4B), Δ*f_n_*= *A* + *Bc*, Δ*D_n_*= *C* + *Dc*. The unit of concentration is mg/mL. The small values of *A* and *C* suggest that both Δ*f_n_* and Δ*D_n_* are proportional to concentration.

| *n* | Δ*f_n_*= *A* + *Bc* | | | Δ*D_n_*= *C* + *Dc* | | |
| --- | --- | --- | --- | --- | --- | --- |
|  | *A* | *B* | *R* | *C* | *D* | *R* |
| 3 | -1.811±0.267 | -1.097±0.005 | 0.9999 | 1.655±0.409 | 1.461±0.007 | 0.9999 |
| 5 | -1.249±0.472 | -0.787±0.008 | 0.9996 | 1.425±0.216 | 0.970±0.004 | 0.9999 |
| 7 | -0.723±0.350 | -0.617±0.006 | 0.9996 | 0.875±0.173 | 0.742±0.003 | 0.9999 |
| 9 | -1.137±0.343 | -0.504±0.006 | 0.9994 | 0.63±0.144 | 0.610±0.002 | 0.9999 |
| 11 | -0.919±0.286 | -0.437±0.005 | 0.9995 | 0.608±0.106 | 0.514±0.002 | 0.9999 |
| 13 | -0.811±0.338 | -0.390±0.006 | 0.9991 | 0.481±0.090 | 0.445±0.002 | 0.9999 |

**S8.** In the case of a shear oscillation, for Zimm and Rouse models, the general methods to describe the dependence of viscosity on shear frequency are, respectively^30^

$\eta(f)=\eta_{\infty}+(\eta_{0}-\eta_{\infty})\frac{\cos[(1-1/3v)\arctan(\omega\tau)]}{[1+(\omega\tau)^{2}]^{(1-1/3v)/2}}$ (S6)

$\eta(f)=\eta_{\infty}+(\eta_{0}-\eta_{\infty})\sqrt{\frac{\sqrt{1+(\omega\tau)^{2}}+1}{2[1+(\omega\tau)^{2}]}}$ (S7)

The form of equation S6 is similar to the Carreau model, which was employed by Munro et al. to describe the shear frequency dependence of PA solution viscosity^1^

$\eta(f)=\eta_{\infty}+\frac{\eta_{0}-\eta_{\infty}}{[1+(\omega\tau)^{2}]^{(1-1/3v)/2}}$ (S8)

The common characters of equations S6 - S8, as shown in Figure S5, are that in the low shear frequency regime (*ωτ* << 1), *η*(*f*) = *η*_0_ and in the high shear frequency regime (*ωτ* >> 1), *η*(*f*) - *η*_∞_ ~ *ω*^α^.


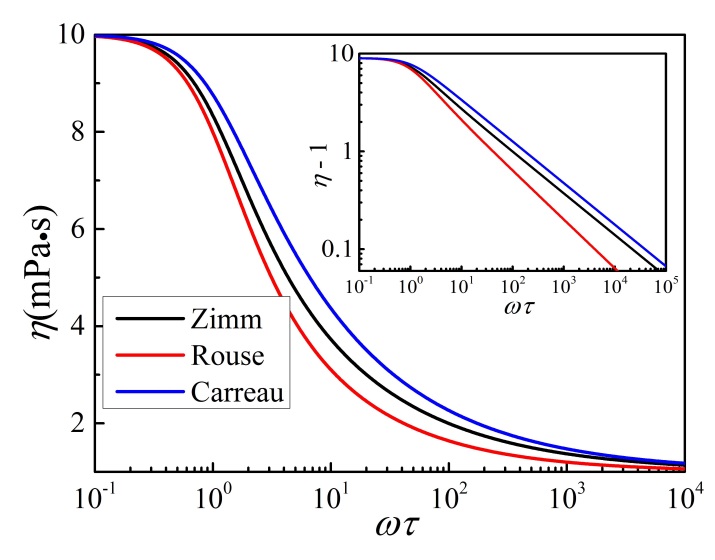


**Figure S5**. Dependences of apparent viscosity on *ωτ*. Inset: double-logarithmic plot of *η*(*f*) - 1 versus *ωτ*. *η*_0_ = 10 and *η*_∞_ = 1 mPa⋅s. The apparent viscosity following Rouse model decreases the quickest and following Carreau equation the slowest.

The calculation results showed that indeed *ωτ* >> 1 (Figure 5). Then the equations S6, S7, and S8 can be replaced by their forms simplified at a high shear frequency condition (equations 1, 2, and 3 in the manuscript), with invariable calculate results.

Additionally, please note that before QCM data modeling, in general a specific relation between apparent viscosity and shear frequency has also to be given. This is because even for the simplest case, a thin homogeneous film, fitting parameters contain four items (density, thickness, viscosity and shear modulus), while number of data points is only two. We would add up to 14 fitting parameters when combing Δ*f_n_* and Δ*D_n_* with *n* from 3 to 13, and assuming that density and thickness are frequency independent, and viscosity and shear modulus are frequency dependent. The data modeling in this case is impossible.

For feasibility, algorithm based on Voight model proposed that both viscosity and shear modulus are frequency independent^1^, and Johannsmann et al. assumed that within a limited frequency range, the dependences are often well approximated by power laws, *G’*(*ω*) = *η*(*ω*)*ω* - *η*_b_*ω* = *G*_0_’ (*ω*/*ω*_0_)*^α’^* , *G’’*(*ω*) = *G*_0_’’ (*ω*/*ω*_0_)*^α’’^*, where *ω*_0_ is an arbitrarily chosen reference frequency, and *η_b_* is the solvent viscosity^31-33^.

It is clear that the cases suggested by Hook et al. and Johannsmann et al. correspond to a small and large values of *ωτ* in equations S6, S7, and S8, respectively.

**References:**

1. Munro, J. C. & Frank, C. W. Polyacrylamide adsorption from aqueous solutions on gold and silver surfaces monitored by the quartz crystal microbalance. *Macromolecules* **37**, 925-938 (2004).

2. Bordes, R.& Hook, F. Separation of bulk effects and bounded mass during adsorption of surfactants probed by quartz crystal microbalance with dissipation: insight into data interpretation. *Anal. Chem*. **82**, 9116-9121 (2010).

3. Alcoutlabi, M. & Mckenna, G. B. Effects of confinement on material behavior at the nanometer size scale. *J. Phys.: Condens. Matter* **17**, R461-R524 (2005)

4. Frank, B., Gast, A. P., Russell, T. P., Brown, H. R. & Hawker, C. Polymer mobility in thin films. *Macromolecules* **29**, 6531-6534 (1996).

5. Semenov, A. N. Dynamics of entangled polymer layers: The effect of fluctuations. *Phys. Rev. Lett*. **80**, 1908-1911 (1998).

6. Koga, T. *et al*. Reduced viscosity of the free surface in entangled polymer melt films. *Phys. Rev. Lett*. **104**, 066101 1-4 (2010).

7. Ellison, C. J. & Torkelson, J. M. The distribution of glass-transition temperature in nanoscopically confined glass formers. *Nat. Mater.* **2**, 695-700 (2003).

8. Roth, C. R., McNerny, K. L., Jager, W. F. & Torkelson, J. M. Eliminating the enhanced mobility at the free surface of polystyrene: fluorescence studies of the glass transition temperature in thin bilayer films of immiscible polymers. *Macromolecules* **40**, 2568-2574 (2007**)**.

9. Zheng, X. *et al*. Reptation dynamics of a polymer melt near an attractive solid interface. *Phys. Rev. Lett*. **74**, 407-410 (1995).

10. Lin, E. K., Kolb, R., Satija, S. K. & Wu, W. L. Reduced polymer mobility near the polymer/solid interface as measured by neutron reflectivity. *Macromolecules* **32**, 3753-3757 (1999).

11. Bodiguel, H. & Fretigny, C. Reduced viscosity in thin polymer films. *Phys. Rev. Lett*. **97**, 266105 1-4 (2006).

12. Zheng, X. *et al*. Long-range effects on polymer diffusion induced by a bounding interface. *Phys. Rev. Lett*. **79**, 241-244 (1997).

13. Priestley, R. D., Ellison, C. J., Broadbelt, L. J. & Torkelson, J. M. Structural relaxation of polymer glasses at surfaces, interfaces, and in between. *Science* **309**, 456-459 (2005).

14. Kanazawa, K. K. & Gordon II, J. G. Frequency of a quartz microbalance in contact with liquid. *Anal. Chem*. **57**, 1770-1771 (1985).

15. Saluja, A. & Salonia, D. S. Measurement of fluid viscosity at microliter volumes using quartz impedance analysis. *AAPS PharmSciTech* **5**, 68-81 (2004).

16. Wang, P., Fang, J. J., Qin, S., Kang, Y. H. & Zhu, D. M. Molecular weight dependence of viscosity and shear modulus of polyethylene glycol (PEG) solution boundary layers. *J. Phys. Chem. C* **113**, 23793-13800 (2009).

17. Wang, P., Fang, J. J., Hou, Y., Du, X. B. & Zhu, D. M. Viscoelastic properties of polyethylene glycol (PEG) boundary layers near a solid substrate. *J. Phys. Chem. C* **113**, 729-735 (2009).

18. Qin, S. *et al*. Viscoelastic signature of physisorbed macromolecules at the solid-liquid interface. *J. Colloid Interf. Sci.* **383**, 208-214 (2012).

19. Keller, C. A. & Kasemo, B. Surface specific kinetics of lipid vesicle adsorption measured with a quartz crystal microbalance. *Biophys. J* **75**, 1397-1402 (1998).

20. Zhang, G. Z. Study on conformation change of thermally sensitive linear grafted poly(*N*-isopropylacrylamide) chains by quartz crystal microbalance. *Macromolecules* **37**, 6553-6557 (2004).

21. Kanazawa. K. K. Gordon II, J. G. The oscillation frequency of a quartz resonator in contact with liquid. *Anal. Chim. Acta* **175**, 99-105 (1985).

22. Wu, B., Wu, K., Wang, P. & Zhu, D. M. Adsorption kinetics and adsorption isotherm of poly(*N*-isopropylacrylamide) on gold surfaces studied using QCM-D. *J. Phys. Chem C* **111**, 1131-1135 (2007).

23. Wu. K. *et al.* Adsorption isotherms and dissipation of adsorbed in its swelling and collapsed states. *J. Phys. Chem. B* **111**, 8723-8727 (2007).

24. Yuan, B., Zhu, T., Zhang, Z. X., Jiang, Z. Y. & Ma, Y. Q. Self-assembly of multilayered functional films based on graphene oxide sheets for controlled release. *J. Mater. Chem.* **21**, 3471-3476 (2011).

25. Zhu, T., Jiang, Z. Y., Nurlybaeva, EI Mi Ra., Sheng, J. & Ma, Y. Q. Effect of osmotic stress on membrane fusion on solid substrate. *Langmuir* **29**, 6377-6385 (2013).

26. Zhu, T., Jiang, Z. Y. & Ma, Y. Q. Adsorption of nanoparticles and nanoparticle aggregates on membrane under gravity. *Appl. Phys. Lett*. **102**, 153109 1-3 (2013).

27. Rodahl, M. & Kasemo, B. On the measurement of thin liquid overlays with the quartz-crystal microbalance. *Sensors Actuat. A* **54**, 448-456(1996).

28. Voinova, M. V., Rodahl, M., Jonson, M. & Kasemo, B. Viscoelastic acoustic response of layered polymer films at fluid-solid interfaces: Continuum Mechanics Approach. *Phys. Scr*. **59**, 391-396 (1999).

29. Pamies, R., Lopez Martinez, M. C., Hernandez Cifre, J. G. & Garcia de la Torre, J. Non-newtonian viscosity of dilute polymer solutions. *Macromolecules* **38**, 1371-1377 (2005).

30. Rubinstein, M. & Colby, R. H. *Polymer Physics* (Oxford University Press: New York, 2003).

31. Johannsmann, D. Viscoelastic analysis of organic thin films on quartz resonators. *Macromol. Chem. Phys*. **200**, 501-516 (1999).

32. Johannsmann, D. Viscoelastic, mechanical, and dielectric measurements on complex samples with the quartz crystal microbalance. *Phys. Chem. Chem. Phys*. **10**, 4516-4534 (2008).

33. Reviakine, I., Johannsmann, D. & Richter, R. P. Hearing what you cannot see and visualizing what you hear: interpreting quartz crystal microbalance data from solvated interfaces. *Anal. Chem*. **83**, 8838-8848 (2011).
